# Micronesian maritime piloting charts as bioimaging proxies for the rescue of cells on the apoptotic trajectory

**DOI:** 10.1016/j.heliyon.2022.e12035

**Published:** 2022-12-08

**Authors:** Vuk Uskoković

**Affiliations:** a Advanced Materials and Nanobiotechnology Laboratory, TardigradeNano, 7 Park Vista, Irvine, CA, 92604, USA; b Department of Mechanical Engineering, San Diego State University, 5500 Campanile Dr., San Diego, CA, 92182, USA

**Keywords:** Anthropology, Bioimaging, Caroline islands, Etak, Ethnoscience, Nanomedicine, Marshall Islands, Mattang

## Abstract

This interdisciplinary study falls within the realm of ethnoscience thanks to its resorting to the scientific methods behind the Micronesian canoe voyaging in search of bioimaging tools for the early prediction of cell fate in response to a therapy. Two distinct indigenous methods for navigation across the ocean were assessed as bridges for correlating (i) the interaction of oceanic swells near atolls with the way microcurrents in the cell culture dish may shape the morphology of cells, and (ii) the spatial arrangement of cultured cells with the canoe voyaging from one island to the next. Both methods effectively predicted the cell fate at early time points in the treatment with superparamagnetic nanoparticles, when the adverse effects were still reversible and not apparent yet at the levels of cell morphology, proliferation rate or confluence. The mattang chart, the most fundamental and theoretical of navigational devices used in the Marshallese seafaring tradition, was used to measure subtle morphological changes occurring in cells due to the treatment. The cells subjected to the treatment were consistently withdrawing their bodies from the areas of intense swell interaction activity on the superimposed mattangs. Given that the cytoskeletal microfilaments defining the features of control cells were largely filling up these areas, this metric proved useful for deducing the course of the treatment at its early stages. The same deduction was proven feasible with the use of a Carolinian navigational technique based on the concept of the etak, in which case the distances traversable between cells in a population subjected to the treatment were divisible to a significantly higher number of etaks than the same distances in the population of control cells. Therefore, treating cells and their nuclei as analogous to Pacific atolls navigable to and fro with the use of imaginary microscopic canoes and the navigational principles native to the Marshall and the Caroline Islands proves as a clever, but also very effective cell fate prediction approach, which various branches of biomedical science could take advantage of. These practical benefits notwithstanding, this conceptual study was performed primarily with a goal to spark the interest in studying these and other ancient ethnoscientific inventions as potential addenda to the broad repertoire of techniques used in biomedical and other sciences to combat some of their greatest challenges.

## Teaser

Anthropology; Bioimaging; Caroline Islands; Etak; Ethnoscience; Nanomedicine; Marshall Islands; Mattang; Wave.

## 1 Introduction

“Could we ever bring them back

Once they have gone?

Oh, Caroline, no”

The Beach Boys, 1966 [1].

Imagine this. You are at the ocean shore, facing the endless body of water in front of you. The day is warm. You decide to enter the water. You swim away from the coast and then stop.

You drift. The swell begins to shift you. Your movements in space become determined by a force greater than you, a force traversing enormous oceanic depths and aerial spaces. The wind wave patterns shaped over thousands of miles in radius are swaying you. You feel infinitely light. And overcome. And somehow free.

Then imagine what it would feel like to miniaturize yourself and take a seat on one of the rafts that traverse the lipid bilayers of the membrane of a cell as it sits attached to the bottom of a cell culture dish. Every once in a while you would jump off this raft into the liquid medium near the membrane and then get back onboard. You might realize that a similar swell felt while drifting near the ocean coast is felt here too, in this microscopic milieu. You may perceive the cell edges sway under the influence of these microstreams, the products of the pressure gradients at the liquid/air interface and around the osmotic barriers conditioning the metabolism of the cell. Just as the swimmer drifts in the ocean, all along with the sand and other solid features of the coastline on a longer timescale, so might the cellular outlines adjust morphologically to the effects of these microcurrents that wash over the lipid bilayer and receptor shores.

What is more, if this sway is realistic, then it must be different for cells submerged at the bottom of a tissue culture dish than it is for cells in their native biological environment, where water that the cells come into contact with is under confinement, having less of its bulk properties and more of the interfacial, liquid crystal ones [2]. Besides, with the daily advances in miniaturized, microfluidic bioreactors, where cells are exposed to complex and oftentimes high-pressure flows [3], vastly different from those present in static culture conditions, it is imperative to understand the effects of such microcurrents on cell behavior. The last few decades in tissue engineering have seen a disproportionate focus on the studies of control of cell fate through the control of properties of solid materials onto which the cells adhere, ranging from the chemical composition [4] to topography [5] to mechanical stiffness [6], leaving the hydrodynamic effects of the fluid medium on the cells understudied in comparison. It is known, however, that the dynamics of the fluid flow through three-dimensional scaffolds has a critical effect on the fate of cells seeded inside them [7] and that structures of the cellular junctions and cytoskeleton can be altered by controlling fluid flow regimens inside the cerebral microvasculature [8], likely affecting consciousness and cognition [9]. Therefore, it is clear that the investigation of the relationship between cell behavior and hydrodynamic properties of the medium in which they grow should be more copious than it is today. Right after this thought, the question of analogy between these microscopic and macroscopic phenomena arises in your head: could there be a single model that describes both of these effects, namely the sway of cells in their liquid medium and the sway of your body on the surface of the ocean? This is a bold question underpinning one central premise of this study.

Then imagine that you are a canoeist navigating through the vast expanses of the open sea from one island in the Pacific to another. You must travel by night because only then could stars be your guide. The stars move across the celestial sphere and as they align with the features of some distant coasts on the horizon, they give you a sign and a wink that you are on the right path. This hypothetic journey on an ancestral boat takes us to the doorstep of the second key premise underlying this study. It rests in the domain of the translational ordering of cells in space and in the question whether there can be a model that correlates a hypothetic journey from one cell to another in the cell culture dish with this sea voyage between two shores dozens or hundreds of miles distant from one another in an ocean brimming with islets and coral atolls.

The interdisciplinary study reported here sets on a voyage to discovery starting from these two basic hypotheses. It falls within the realm of ethnoscience thanks to its resorting to the scientific tradition of mostly extinct local indigenous cultures, in this case of Micronesian canoe voyaging, in search of tools for solving the actual puzzles in biomedical research. By showing an interest in the traditions of knowledge emerging from the less technologically advanced societies, ethnoscience implicitly challenges the elitism tied to the scientific practice in the west. Ethnoscience, as such, is intrinsically political and very often, as is the case with this particular study, it sheds light on the imperialistic political backdrops of science. As per the motto that “scholarship follows the colonial flag” [10], these political roots of science can help it grow, but when science finds itself tangled in a complex web of geopolitical interactions, these roots can also suppress its growth or eradicate it completely. Such was the case with numerous traditional scientific practices that disappeared due to colonization, globalization and other means of disseminating the principles of universalism and uniformity. Countless scientific models that could have grown to equally influential streams of knowledge as those dominant in the western world today were slayed during the course of the history by the sword of nasty politics. Some of them have never been noted in the literature and some of them have, albeit with very little or none of the living practitioners. Therefore, the past accounts instruct us that not the best, but the most powerfully backed epistemologies proliferate by feeding on their less well politically fortified counterparts. Once this historical principle is understood, western science becomes seen as only one out of an infinitude of possible ways of scientifically approaching the physical reality, owing its global dominion to a large extent not only to its abstract subtleties, but also to the political support throughout the centuries. Reiterating its premises is, consequently, analogous to perpetuating the detrimental effects entailing the proliferation of a single worldview at the expense of the eradication of countless other, equally relevant ones. In contrast, excavating the long-forgotten scientific concepts may prove to be analogous to expanding the epistemic bases of humanity, deepening its cultural roots and enhancing its sustainability.

In search of these ancestral models, we will first enter the world of a native Lollelaplapian youth instructed how to navigate the waters around the many atolls and islands of the Micronesian archipelago known today by the name of the Marshall Islands. At first, before he is allowed to take hold of the sails or taught how to comprehend the navigational charts, he spends days, weeks, months and perhaps years lying down in the bow of an outrigger canoe, feeling the swell and the currents. Then he is shown how to sense the finest ocean wavelets and correctly estimate their strength, their direction and their interference with one another [11]. Acquisition of the most sophisticated skills in ancestral traditions was tied to learning specific meditation techniques [12], and so is likely to have been the case here. In Debussy's *Submerged Cathedral*, the sense of floating on the sonorous waves owes itself to the lack of key in the composition and, likewise, it is conceivable that the youth was first taught how to liberate his mind from anchorages onto any abstract grounds so as to be able to float perfectly and perceive the finest wavelets swaying him. Only after he has mastered this art of sensing the movement of the waves is he permitted to proceed to the next step, which involves the real navigation in correspondence with the mental images of the charts mapping the ocean. After this journey into the world of the indigenes of the Marshall Islands, we will travel about 1300 miles to the west, to the Caroline Islands, where we would get acquainted with the traditional navigational technique based on following the alignment of sometimes real, sometimes mysterious or purely fantastic reference islands with the stars, and test if correlations could be made between this route-finding method and the spatial arrangement of cultured cells in response to a therapy. These two ancestral navigational methods, one natively Marshallese and one Carolinian, correspond, respectively, to the two aforementioned basic premises underpinning this study.

Pacific Islanders are known for being the sources of versatile ethnoscientific concepts [13]. Many of them have been the subjects of intense anthropological research because of belonging to distant pasts and having a very small population of contemporary practitioners, which are more often than not on the brink of extinction. Such exactly is the case with the native Marshallese and Carolinian arts of navigation that are at the foci of this interdisciplinary study. The indigenous seafarers from Oceania came up with a number of maritime inventions, including the crab claw sail on the ancient Polynesian outrigger canoes [14], the coir rope fabrication methods developed in Fiji [15], and the Ifalik and Puluwatan star compasses [16, 17] invented in the Caroline Islands. However, what is unique about the Marshallese school of wayfinding is that while all the other Pacific islanders used the wind and the stars to orient themselves at open sea, the Marshallese built their navigational system around sensing the disruptions of swells and currents by the atolls and islands dispersed across the ocean. Sadly, however, this non-instrumental, nonmetric and to a large extent nonvisual art of wayfinding has disappeared and there are only rare surviving elders who remember it. Because of the numerous waves of colonization, the interest in these traditional methods of navigation began to decline in favor of more modern technologies already by the late 19^th^ century, and in the mid-20^th^ century, around the time of the Bikini atoll nuclear testing, the last traditional seafaring teaching camps in operation in the Islands closed. As a result, some of the most elementary aspects of this unique navigational method are currently subject to dispute, carrying multiple possible meanings based on their actual interpretation. In fact, even the ancient name the native Marshallese ascribed to their lands and seas is subject to a dispute, with words such as Lollelaplap and Aelon kein ad being the contenders, but also dismissed by many as recent artificial creations in the effort to shed the colonial past [18].

Be that as it may, the story behind the decline of the indigenous sciences in what is today known as the Marshall Islands, after 2000 years of continuously harboring human settlements, is the one underlain by the erroneous imperialistic premise that one culture fits all, leading to globalization and propagation of uniform worldviews and modes of being. A similar, albeit somewhat less dramatic and eventful fate contributed to the disappearance of the ancient navigational technique based on the concept of the mystical reference islands in the Carolines. Like the Marshall Islands, the Carolines underwent successive waves of colonization, gradually losing their indigenous identity and taking on more advanced maritime technologies than the traditional. The comfort of relying on modern navigational systems and means of transportation compared to years of apprenticeship needed to get skilled in canoeing between islands without compasses or charts has taken its toll and, like the Marshallese art of navigation, so has the Carolinian been descending into an ever deeper obscurity with each passing year.

In this ethnoscientific study, the revival of these two ancient spatial orientation methods is attempted via their semantic cross-fertilization with the actual problematics in cell biology. Specifically, these ancestral wayfinding techniques were used to assess the morphological and cell spacing indicators of the cell fate in response to a therapy delivered in the form of inorganic superparamagnetic nanoparticles. Because of their small size, high surface-to-volume ratio and good dispersibility, inorganic nanoparticles are being intensely researched for delivering various forms of stimuli to the cells [19, 20, 21, 22], with the goal of controllably altering their fate. Here, the nanoparticle therapy was designed so as to elicit a gradual decrease in the viability of cancer cells, which is at its earliest time points accompanied with neither gross morphological deformities nor reductions in cell density that would serve as blatant visual cues of the fate awaiting the cells in the near future. Catching this early moment when the morphology of the cells or their arrangement in space may indicate that they have embarked on the dying trajectory is crucial, as it enables the saving of the system with an appropriate therapeutic action delivered timely. In this study, the detection of this critical crossroad where viability transforms into unviability is being achieved by comparing the single cell morphology and the arrangement of cellular congregates with the seafaring devices and schemes invented and relied upon by the indigenous Marshallese and Carolinians, respectively. This attempt to rescue a biological system destined for liquidation with an ancient maritime orientation technique ties with the bonds of analogy to the fate that awaited many indigenous Pacific island cultures, the Lollelaplapian and the Carolinian included, which these words and lines, similarly, aspire to save from descents to oblivion.

Lollelaplapians of the mid-19^th^ century might not have recognized the imminent peril of their culture when the first German trading company opened on one of the atolls by Adolph Capelle in 1864. This resulted in the annexation of the Islands by Germany in 1885 and subsequent colonization, first by Japan in 1914 and then by the United States in 1944, which continues to use the largest of the Marshall Islands, Kwajalein, as a military base for missile testing, having prompted a series of evacuations, emigrations and other forms of disruption of the traditional social fabric throughout the last century and a half. Not only have the atomic bombs detonated on the Marshall Islands between 1946 and 1958 - amounting to 1.7 Hiroshima bombs each day for 12 years [23] - taken a direct toll on the health of the Marshallese [24], with some of the atolls, such as Bikini, Enewetak and Rongelap, having higher concentrations of some of the radioactive elements, such as ^239/240^Pu, than the exclusion zones at Chernobyl and Fukushima today [25], but overpopulation caused by migration to some of the islands, such as Ebeye, to take advantage of the job opportunities at the United States military base at Kwajalein have led to the poor living conditions and rises in diabetes, tuberculosis and other infectious diseases, following in step the deterioration of traditional cultural values and skills, including the ancient art of maritime navigation. Dependency on the foreign aid to compensate for the damage done by the nuclear testing and the mass migration to the United States since the late 1970s [26], [27] have further contributed to the abandonment of the old culture for a western lifestyle that has, in fact, never materialized. The 19^th^ century Carolinians must have been similarly oblivious to the pending disappearance of their ethnic cultures in the wake of similar waves of colonization as those that affected the Marshall Islands, first by Spain, then by Germany, Japan and, finally, the United States. But had the Lollelaplapians drifting dreamingly in the bows of their canoes sensed this wave of colonization coming, the way they could sense the subtlest wavelets in the water, perhaps this traditional culture would be living today and not be confined to history books. Likewise, had the indigenous Carolinians sensed that the vanishing islands that they used as reference points in their voyaging were a sign of gloomier things to come, namely the vanishing of their ancient cultures and their arts [28], they might have approached the inflow of change from abroad with a different attitude. Curiously, it is the seafaring tools invented by these two virtually extinct cultures that are explored here as bioimaging devices for rescuing cells from adverse treatments before the wave of woe sweeps them and it becomes too late.

## 2 Experimental part

### 2.1 Cell culture

K7M2-pCl murine osteosarcoma cell line was purchased from the American Type Culture Collection (Manassas, VA, USA). Cells were grown to confluence before being plated on 12 mm circular glass cover slips or in 48-well culture plates. Cells were maintained in Dulbecco's modified Eagle's medium (DMEM) supplemented with 10% fetal bovine serum (FBS) and 1% antibiotic-antimycotic (Life Technologies, Carlsbad, CA). The medium was replaced every 48 h, and the cultures were incubated at 37 °C in a humidified atmosphere containing 5% CO_2_. Upon reaching the confluence, the cells were detached from the cell culture flask surface using 0.25 wt.% trypsin, washed, centrifuged (1000 rpm × 3 min), resuspended in fresh media and subcultured. The cultures were regularly examined under an optical microscope to monitor growth and possible contamination.

### 2.2 Nanoparticle synthesis and characterization

Composite nanoparticles consisted of iron oxide cores, silica shells and carbon crusts and were modeled after the stratified structure of the planet Earth [29]. Their synthesis using a hydrothermal method and characterization using a variety of physicochemical techniques are described in more detail elsewhere [30]. Briefly, iron oxide nanoparticles were precipitated from aqueous solutions containing 10 mM FeCl_3_, 5 mM FeCl_2_ and 0.1 vol.% Triton X-100 using a mixture of ammonia and NaOH. The resulting dispersion of the nanoparticles continued to be stirred and aged at 80 °C for 1 h. A 1:1 mixture of tetraethylorthosilicate (TEOS) and (3-aminopropyl)triethoxysilane (APTES) was then added to the suspension to deposit the silica layer. Carbon coating was deposited in a hydrothermal reactor (Parr), using citric acid as the carbon coating precursor. The reaction was run at 200 °C for 1 h. The suspensions were concentrated by centrifuging in Amicon Ultra-4 centrifugal filter tubes (Ultracel 100-K, 100,000 M_w_) to yield stable ferrofluids.

### 2.3 Nanoparticle characterization

High resolution transmission electron microscopy (HR-TEM) analysis was carried on a JEOL 2100F microscope equipped with a Schottky-type field emission source and a cryo-polepiece operating at 200 keV. All images were recorded using a Gatan OneView camera with the point-to-point resolution of 0.26 nm, the lattice resolution of 0.1 nm, and the information limit of 0.124 nm. X-Ray Diffraction (XRD) was carried out on a Bruker D2 Phaser diffractometer using polychromatic Cu as the irradiation source. The K_β_ line was stripped off with an inbuilt filter, whereas the K_α2_ line was stripped off manually. The step size was 0.01 °, with 1 s of the specimen irradiation per step.

### 2.4 Cell viability assay

Near confluence, K7M2-pCl cells were divided to two groups: the control one and the treatment one. The cells in the treatment group were treated with 5 mg/ml of composite superparamagnetic nanoparticles administered to the medium as a stable colloidal suspension and incubated at 37 °C and 5 % CO_2_ for 24 h. The dose-response relationship is dependent on the physicochemical characteristics of the nanoparticles, the cell type, the passage number, the confluence, and the incubation time. The viability may plateau for certain concentration ranges, but is overall inversely dependent on the nanoparticle dose [31]. Under the conditions reported here, the loss of viability was partial and, thus, ideal for the purpose of this study. The cell viability was measured after the incubation time of 24 h using the 3-[4, 5-dimethylthiazol-2-yl]-2,5-diphenyl tetrazolium bromide (MTT) assay. A 12 mM MTT stock solution was first prepared by adding 1 ml of sterile phosphate buffered saline (PBS) to a 5 mg vial of MTT and vortex-mixing to ensure complete dissolution. After 24 h of incubation with the nanoparticles, cells were washed with PBS and 275 μl of 1:10 vol./vol. MTT/media was added into each well. After 4 h of incubation at 37 °C, 210 μl of the solution was removed and 125 μl of dimethyl sulfoxide (DMSO) was added to each well. The plates were placed in a 37 °C incubator shaker at 120 rpm for 30 min before measuring the absorbance at 570 nm using the BMG LABTECH FLUOstar Omega microplate reader. Viability was expressed in percentages and normalized to the absorbance of the negative control, containing the growth medium alone. To account for the effect of nanoparticles *per se* on the absorbance in the lysate, the absorbance of wells containing the growth medium and the nanoparticles, but no cells, was subtracted from that of the cells treated with the nanoparticles.

### 2.5 Fluorescent cell staining

Control and nanoparticle-treated cell cultures on glass cover slips were fixed with 4% paraformaldehyde, then washed with the wash buffer composed of 0.1 wt.% Triton X and 0.1 wt.% bovine serum albumin in PBS, and then stained with 0.2 ml per well of the staining solution containing 1:4000 vol./vol. AlexaFluor 568 phalloidin as a staining reagent for f-actin microfilaments and one drop of NucBlue ReadyProbes as a staining reagent for the cell nucleus. These reagents stained the cell nuclei in blue and f-actin microfilaments in red. Fixed cells were incubated at room temperature for 2 h before being mounted and cured with the ProLong Diamond antifade mounting agent. Fluorescent cell images were acquired on a Nikon T1-S/L100 confocal optical microscope and analyzed for cell morphology and pairwise distances between cells using ImageJ (NIH, Bethesda, MD).

## 3 Results and discussion

### 3.1 Nanoparticles and the cell treatment

**Figure 1:**
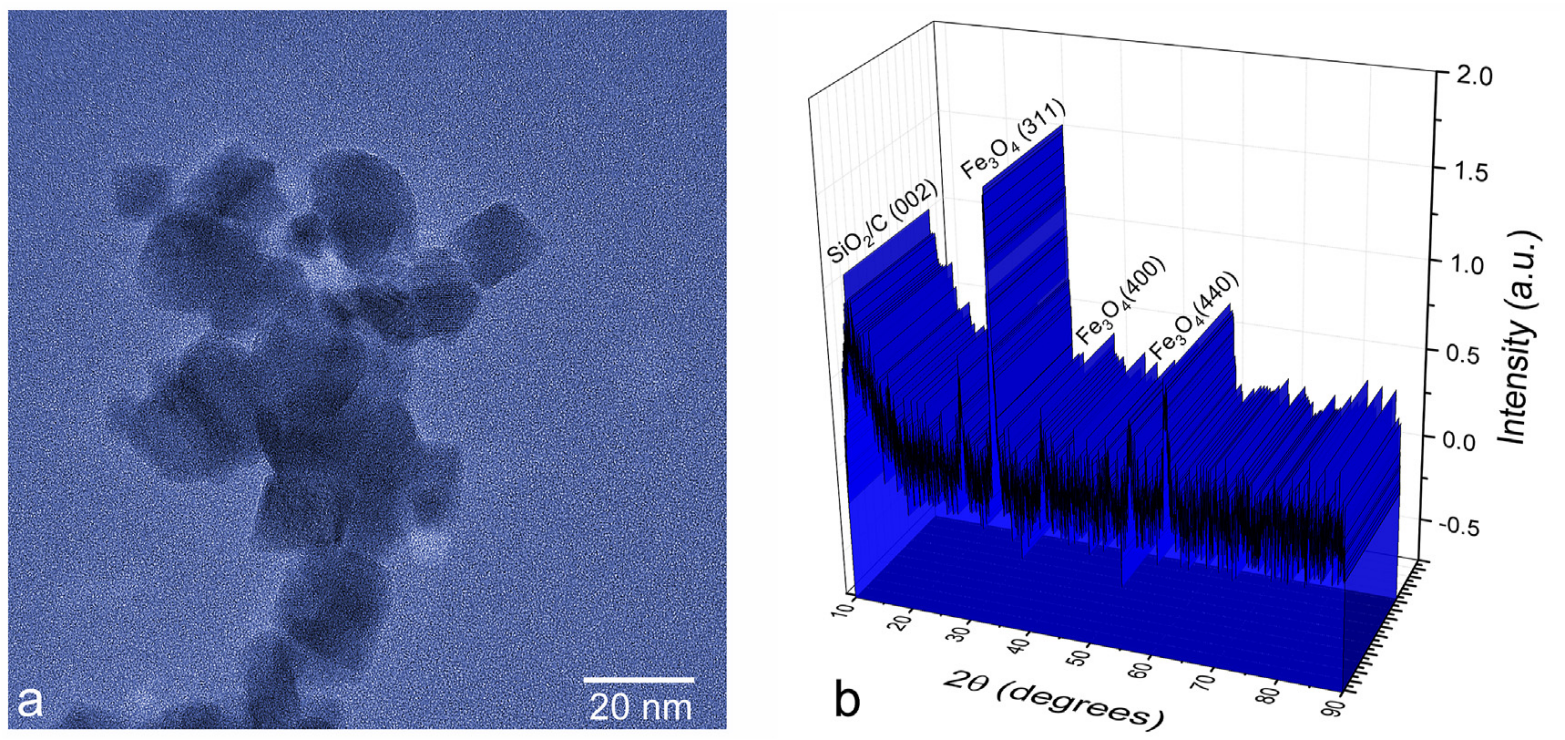
A representative transmission electron micrograph of the composite superparamagnetic nanoparticles (a) and the corresponding X-ray diffractogram denoting the major reflections with their corresponding Miller indices for each of the phases (b).

A representative transmission electron micrograph of nanoparticles used in the cell treatments is shown in Figure 1a, demonstrating the presence of ultrafine and cuboid particles. The particle sizes averaged at 10–15 nm and their sharp edges allowed them to minimize the thickness of the so-called “dead” surface layer resulting from spin canting effects common on the convex surfaces of spheroid particles. The preservation of high crystallinity all the way from the bulk of the particles to their surface explains a relatively high magnetic moment exhibited by them in an external field [32]. The dark, visible portion of the particles shown in Figure 1a corresponds to their superparamagnetic iron oxide cores, while the atomically thin silica and carbon coating appears in the form of a slight chiaroscuro surrounding them. The presence of these two surface components preventing the superparamagnetic cores from agglomeration and phase segregation in water was confirmed in a diffractometric phase composition analysis. The XRD pattern of the nanoparticles is shown in Figure 1b, demonstrating a complex phase composition. Expectedly, the most crystalline component was that of iron oxide taking the form of magnetite/maghemite, while silica and carbon were both amorphous and detected as characteristic diffuse low-angle reflections. The nanoparticles were previously shown to reduce the viability of a number of different cancer cell lines, including glioblastoma and osteosarcoma [33], for which reason they were chosen here to induce a mildly adverse effect on bone cancer cells.

**Figure 2:**
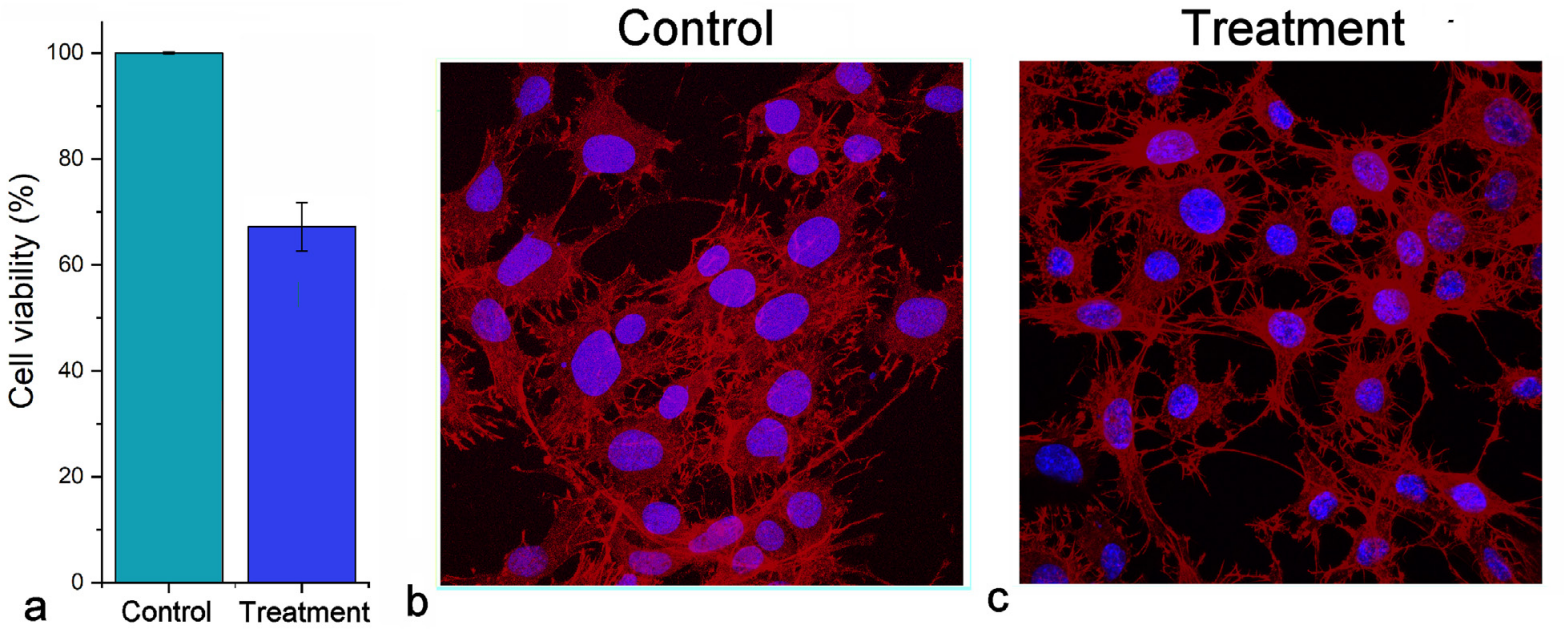
Viability of K7M2-pCl osteosarcoma cells incubated overnight with the superparamagnetic composite nanoparticles relative to the viability of the control K7M2-pCl cells (a) and the optical fluorescent micrographs of the two cell populations, control (b) and treated (c), showing imperceptible differences in morphology or cell density at this early of the stage in the treatment. Cell viability data in (a) represent averages, while error bars represent standard deviation. Cell nuclei in (b) and (c) are stained in blue, while cytoskeletal microfilaments are stained in red.

The outcomes of this treatment after an overnight incubation are shown in Figure 2a. The viability of the cells decreased mildly due to the treatment, while the optical observation of the cells after staining for f-actin cytoskeletal microfilaments and the cell nuclei demonstrated no difference between the untreated control cells (Figure 2b) and cells subjected to the treatment (Figure 2c). The viability testing based on measuring the mitochondrial activity, the results of which are shown in Figure 2a, is a timely process, taking 4–6 h and requiring the lysis of the cells. Most other tests assessing for the cell fate require a similar invasion and destruction of the native cell structure to reach the molecular targets that serve as markers of the trajectory on which the cells are in response to the treatment. Some of such toxicological tests include those based on measuring the luciferase activity to assess the ATP activity, 5-bromo-2′-deoxyuridine assay measuring the DNA synthesis, immunohistochemistry tagging proteins selectively expressed either during the proliferative, S, G2 and M phases of the cell cycle, or the non-proliferative, G0 and G1 phases, but also protein expression analyses such as the western blots or gene expression analyses such as quantitative polymerase chain reaction (qPCR), DNA microarrays, serial analysis of gene expression (SAGE) making use of 3′-end biotin-labeled cDNA, or northern blots [34]. Moreover, measuring cell death at this early of a stage in the treatment, when no cells have begun to leak dead cell-specific proteases, such as lactate dehydrogenase, would not give any meaningful insight. Using simple optical observations of cells can be a noninvasive process if only the right models are developed to magnify the differences in cell morphology and the translational order inherent to intercellular spacings invisible to the naked eye. The search for such models in the ethnoscientific domain led the searcher to the ancestral Micronesian methods for navigation across the Pacific Ocean.

### 3.2 Correlation with the Marshallese mattangs

The most basic device used to instruct the Marshallese mariners in training how to navigate the waters around the coral atolls is a stick chart assembled from palm ribs, coconut leaves, banana fibers, pieces of corals and an occasional seashell or two, called a mattang. Mattang is not a map in the common sense of the word, but rather an idealized and generalized conceptual scheme used to transmit the basic ideas about the swell interaction and, thus, navigation at sea. Thanks to this unique conceptual character, it holds an important scientific and historical value rather than a purely cartographic one. The riders of Marshallese canoes and sailboats would rely on the mental image of the mattang chart in judging about the interaction of swells and navigating the open waters extending over almost a million square miles. The mattang, however, because of its generic character, proves useful only as the most basic educational device. It would instruct the mariners about the fundamental concepts behind the swells and their interactions, while its variants applying directly to particular islands or atolls would be the charts used in real navigation. Even then, though, no charts but those engraved in the memory of the navigators were used onboard. Moreover, all these specific charts could be traced to the most elementary of them: the mattang.

**Figure 3:**
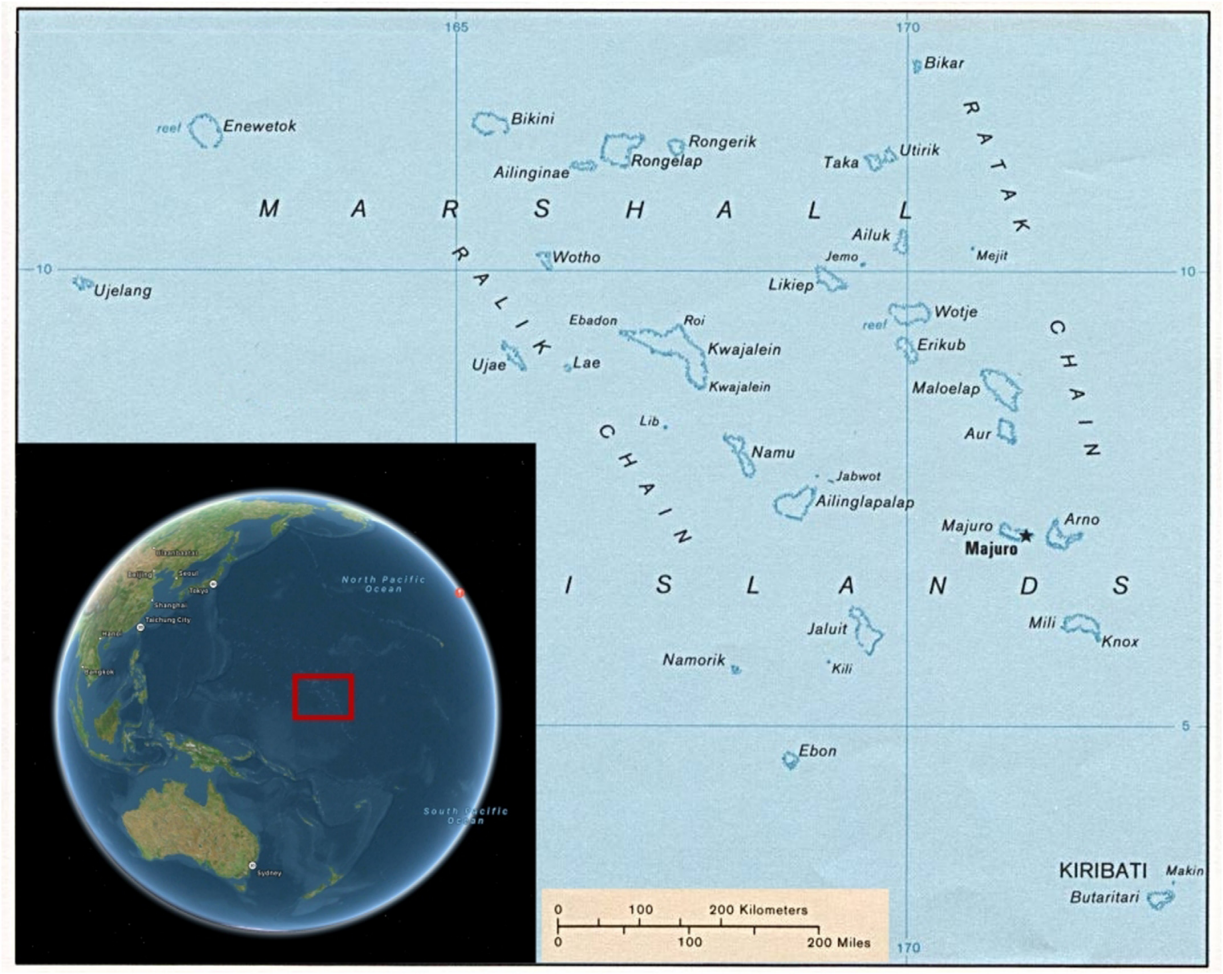
Map of the Marshall Islands and their two main coral chains: Ralik and Ratak. The inlet shows the location of the islands on the globe with respect to the Eurasian and Australian continents.

These more specific charts are called meddo when applying to selected spatial ranges within the Marshall Islands archipelago or rebbelith when mapping either the entire archipelago encompassing 29 coral atolls and 5 coral islands extending over circa 700,000 square miles or any of its two main atoll chains, namely Ralik and Ratak (Figure 3). Historically, most voyages were taking place within the Ralik coral chain because a single chiefdom governed it, as opposed to the Ratak chain, which was divided among various chiefdoms that were frequently at war with one another [35]. Almost all of the atolls in the archipelago were inhabited; voyaging between them, alongside preparing for the voyages, was noted by the missionaries of the 19^th^ century to have taken a considerable portion of time in the life of the Marshallese islanders [36]. For some of these atolls, the distance to the nearest neighboring atoll is relatively low, as it is the case, for example, with Taka and Utrok atolls on the northernmost bounds of the Ratak chain, which are only a few miles apart. For others, however, the distances can be very large. For example, the distance separating the two atolls that were the sites of the nuclear testing by the United States army, namely Bikini and Enewetak, is 216 miles. In fact, the nearest neighbor distance for a Marshallese atoll on average is around 100 miles, which is significantly higher than the horizon distance of 3 miles. What is more, the islets of the atoll are very low, with palms and coconut treetops often being the highest peaks on them [37], which makes any navigation guided solely by vision even harder. Therefore, lest the mariners risked of getting lost in the vast expanses of the Pacific, the use of a precise navigational system that ensured finding an atoll traveled to within such enormous ranges of open sea without the ability to rely on visual cues was absolutely vital.

While meddo and rebbelith charts had a greater practical significance for navigation than the mattang and, unlike the latter, can be called maps in the real sense of the word, the meaning of the Marshallese mattang extends beyond that of the Micronesian archipelago and speaks about a nonmathematical ethnic science that is vastly different from the western science and its mathematical abstractions. It is a science where the haptic, visual, kinesthetic, vestibular, proprioceptive and intuitive components of sensory information processing were all combined into a holistic cognitive output, with the resulting lines on the charts representing neither waves nor currents nor swells nor directions of travel *per se*, but rather all of these combined [38]. Although it is tempting to assume that the Marshallese must have had as many words to describe waves as the Inuit have to describe snow, this appears not to be the case, given the relative succinctness of the vocabulary of both Marshallese dialects and the fact that the wave movement sensations, albeit indisputably versatile, were mostly responded to gesturally, in an action-oriented manner [39]. Still, considering that their navigational method focused almost entirely on the movement of the water, it can be considered methodologically deeper, if not more intuitive, than the seafaring orientation methods devised and used in the Old World, which combined the alignment with the Sun and other stars, the shadows cast by the sails and the vessel, direction of the winds, coastal feature recognition, and other clues. This focus on a single source of orientation signs, namely water, among the traditional Marshallese mariners makes their navigational method fundamentally different from the multiplicity of sources, including wind, stars, water and sound, used by Phoenicians, Carthaginians, Vikings and other ancient seafarers.

**Figure 4:**
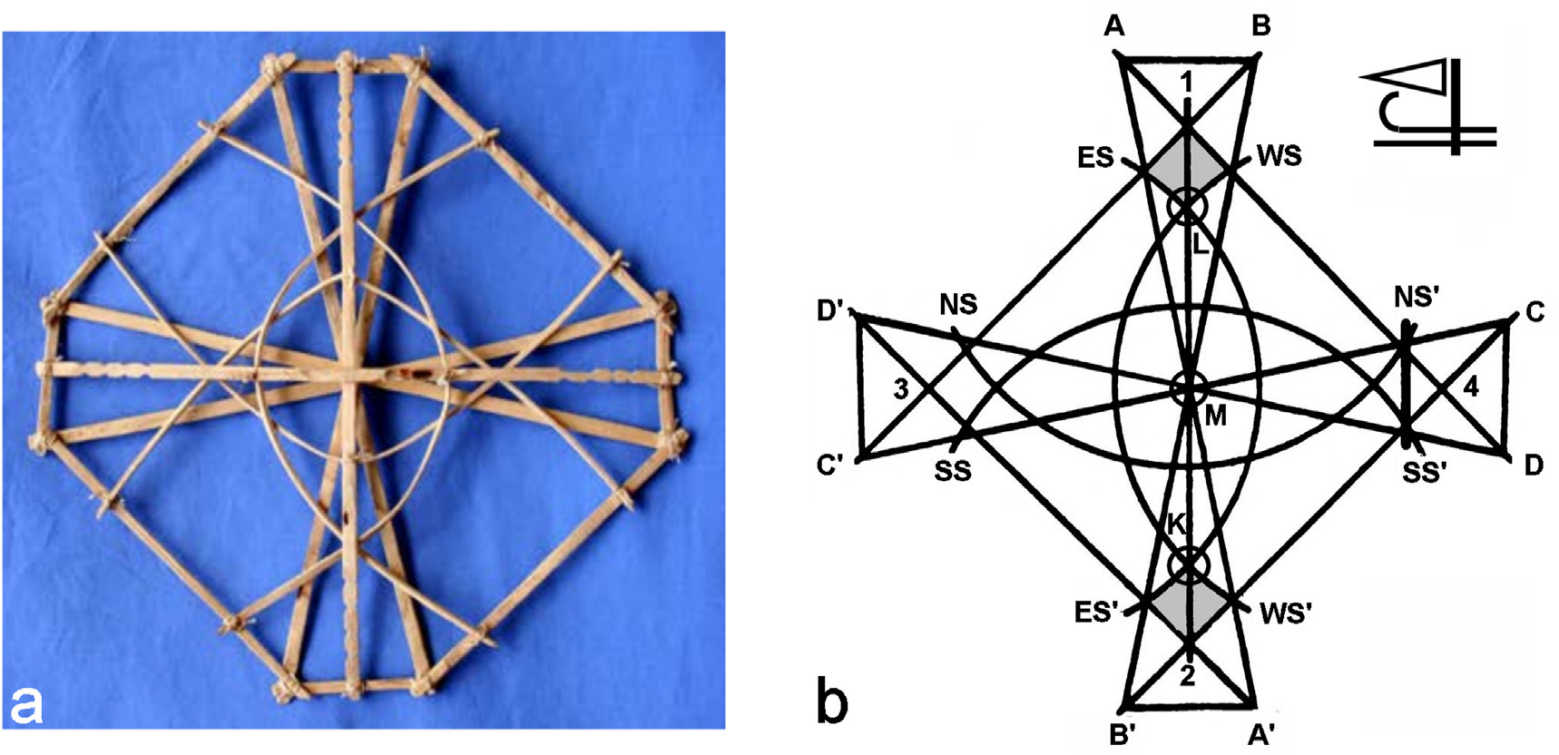
A real-life mattang stick chart assembled from the aerial roots of pandanus (a) and the diagrammatized version of it (b). The mattang is used by the Micronesian canoers to navigate the superimposing swells coming from four cardinal directions and reach the atoll M at the center of the map (b). Subfigure (a) is reprinted with permission from Ref. [41].

A typical mattang is assembled from interlocking sticks, as shown in Figure 4a, and is sizable, having anywhere between 0.6 and 1.2 m in diameter. Intrigued by this device, the Hawaiian missionary, Luther H. Gulick was the first to mention it in a published report from 1862, but could not get any information about its meaning because the islanders kept it a guild secret. A few decades later, the German naval captain Winkler managed to convince the islanders in the value of sharing the principles behind the mattang chart with the world and transcribed it into a diagram [40], like the one shown in Figure 4b. It was in this form that the mattang was correlated with the morphological features of cells in culture. The central point in the mattang, M, is where an out-of-sight atoll that a mariner tries to reach is located. The atoll is surrounded by four swells, each coming from a specific cardinal direction. Thus, for example, the triangle 1-2-3 depicts the western swell, which refracts as it approaches the toll and gets curved, forming the WS-WS′ curve, with WS standing for “western swell”. The same effect is observed for the eastern, the northern and the southern swells, forming ES-ES′, NS-NS′ and SS-SS’ curves around the atoll, respectively. All four swells bend around the atoll and each swell crossing the swell from the direction opposite to it forms nodes of intersection called bot (“knot” or “node”), which are of critical importance for navigation because they define the most convenient channel, a.k.a. okar (“root”), leading the navigator to the island. Two of such bot points where the easterly and the westerly swells meet in Figure 4b are L on the north side and K on the south side, with the lines L-M and K-M forming the two okar channels, the former approaching the atoll M from the north and the latter approaching the atoll M from the south. Here it should be noted that the crossing angles change as the curvatures of the swells change, meaning that okar is a straight line only on this idealized map, whereas in reality it changes the direction and curvature depending on the nature of the interacting swells.

The wind in the mattang chart is depicted as blowing from the east, *i.e.*, perpendicular to the NS’ – NS line, streaming in the westward direction toward the atoll (Figure 4b). Approaching the atoll directly from the leeward point 3 is not convenient, as it would necessitate a great degree of tacking, that is, alternating between sailing in the directions of 45–65 ° with respect to the wind, to compensate for the effects of the wind blowing directly into the sails. Equally, sailing to the atoll directly from the windward point 4, with the wind in one's sails, is discouraged because of the chance that the full force of the wind would swing the sail across the hull. For this reason, when coming from the east or west, the mariner sails at an angle of at least 15–20 ° with respect to the east-west direction of the wind. When coming from the east, it is, therefore, sailed at an angle until the point in the windward zone is reached whereat the eastern swell meets either its reflection from the atoll or the western swell refracted from the atoll. These two points are the most critical points in the mattang diagram, but they come with a dose of ambiguity. For, the shape of the eastern swell curving around the atoll (ES-ES′) can have the same shape as its reflection from the atoll in the eastward direction, for which reason it can be suspected that both of these key points are designated with a single one in the diagram, even though in reality they correspond to two distinct locations in the ocean. Correspondingly, the point of meeting of the eastern swell and its reflection from the atoll may not be marked, lying approximately halfway between the points marked with WS and NS′ on the northern windward side and between the points marked with WS′ and SS′ on the southern windward side, but it may also be identical, at least in the abstract metrology of the mattang, to the point marking the meeting of the eastern and the western swell, namely WS on the northern side and WS′ on the southern side, or, more precisely, the points of intersection of WS-WS′ and A-D in the north and of WS-WS′ and C–B′ in the south. In both cases, the canoer is expected to recognize this crossing point with his immaculate wave-sensing skills and then make a sudden 45 ° turn until the points K or L are reached, depending on whether he approaches the atoll from the south or from the north, after which the line connecting 1 and 2, a.k.a. okar, is reached, and followed directly to the island. For these reasons, the ideal approach is made from the northward and the southward directions when the dominant swells are easterly or westerly, and the strategy is reversed when the swell directions change. Once the okar is reached, sailing to the center of the map, where the atoll is located, involves the traversal of specific points along the vertical stretch extending from north to south where the swells and their countercurrents meet. Although sailing across the okar is often imagined as sailing along a direct, linear course, in reality this involves sailing in a somewhat zigzag fashion, from one meeting point, a.k.a. bot, to another.

Therefore, the most critical area in the course of navigation is that where the main swell, a.k.a., rilib, typically coming from the east, meets the swell directly opposite to it, a.k.a. kaelib, typically coming from the west. This area where the diametrically opposed swells meet and intersect at almost right angles corresponds to the square-shaped areas on the northern and the southern ends, which are colored grey and whose sides are 1-ES-L-WS and 2-ES′-K-WS′, respectively. The side of this area in the eastern swell on the northern side, ES - L, is also known as rolok, meaning “something lost” or “plunge into the sea”, while its mirror line on the southern end, ES′-K, is known as nit in kot, meaning “a hole” or a “cul-de-sac”. As far as the complementary sides of this square-shaped area for the western swell are concerned, both the lines WS-L and WS′-K are called jur in okme, meaning “stakes”. As in accordance with their etymology, these four key lines of paramount importance present the demarcation lines between getting lost and finding the way to the atoll. For example, when searching for the atoll from the north, one is expected to sail between rolok and jur in okme all until the okar (point L) is found, after which the course toward the atoll is, more or less, straightforward. Likewise, when approaching the atoll from the south, one is expected to sail between nit in kot and jur in okme. Failing to locate this critical area in the ocean predisposes one to become a “trapped bird” in the Marshallese language and enter the crazed state of mind a.k.a. wiwijet, resulting in a sail in the wrong direction, without possibly ever finding land again. It is this small and extraordinarily important region in the mattang that is at the focus of the herein described attempt to utilize this ancient chart as a tool for modeling cell behavior.

**Figure 5:**
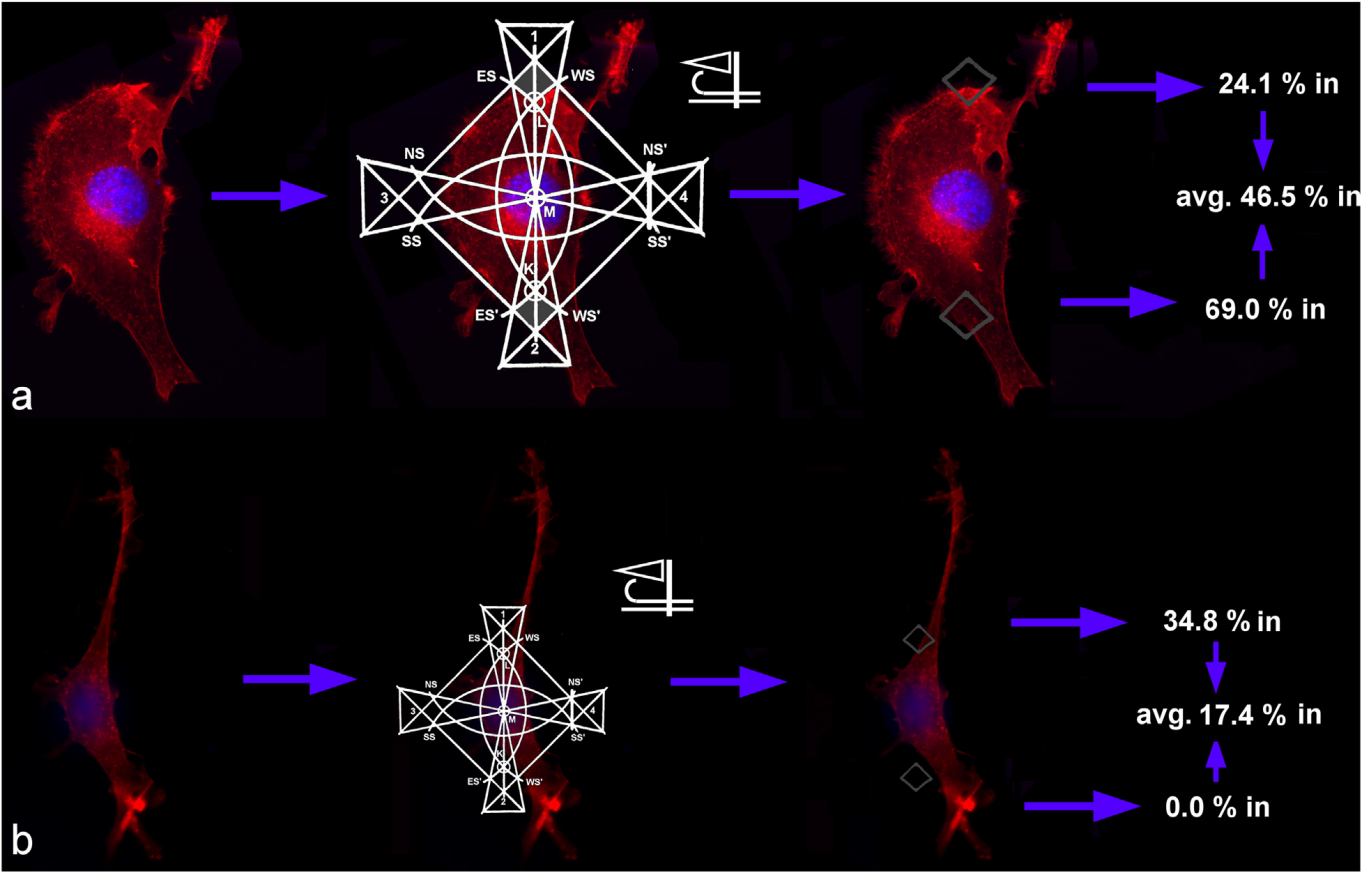
Illustration of the method used to correlate the Marshallese mattangs with the morphology of one control cell (a) and one treated cell (b). The method is based on measuring the proportion of the jur in okme squares lying within the boundaries of the cells oriented windward with respect to the easterly swell. In the control cell image comparison (a), 24.1 % of the surface of the upper jur in okme square, whose edges are, clockwise, 1, WS, L and ES, overlaps with the cell, whereas 69.0 % of the surface of the lower jur in okme square, whose edges are, clockwise, K, WS′, 2 and ES′, overlaps with the cell, yielding the average of 46.5 %. In the treated cell comparison (b), 34.8 % of the surface of the upper jur in okme square and 0 % of the surface of the lower jur in okme square overlap with the cell, yielding the average of 17.4 %.

To analyze the correspondence between the cell images and the Micronesian mattangs, the latter were superimposed over the images of individual cells belonging to the two sample groups, one of which was the control and the other one of which was subjected to the treatment. A special attention was paid to the adjustment of the cell orientation and size with respect to the overlain mattangs. As for the orientation, it was assumed that an analogue of the wind, blowing the cell protrusions toward one end of the mirror plane, is at work at the cellular level, too. Therefore, the cell was oriented in such a way as to suggest its being deformed by the incoming wind from the east (Figure 5). As for the size, the cell nucleus was made to fit the area bordered by the SS-SS′-NS′-NS rectangle, with M in the middle (Figure 5). Then, the proportion of the area of the cell fitting the two square-shaped areas bordered by jur in okme and rolok/nit in kot was measured and compared across samples. For the sake of clarity, these square-shaped regions of the mattang superimposed over the cells will be called from here on, simply, jur in okme squares. A crucial challenge encountered during the implementation of this superimposition method was that of the correct positioning of mattangs over individual cellular images. Because of the fine variations in this positioning and a relatively broad margin of error in the portion of the aforementioned jur in okme squares overlapping with the cell contours, a sufficient number of cellular replicas had to be utilized in the measurements to gain a statistically reliable comparison. An image recognition algorithm that would compute the most optimal size and angle of the mattang with respect to the cell, perhaps with the help of fractional calculus [42] or non-negative matrix factorization [43], would be a step forward compared to the manual positioning thereof implemented here, with the caveat that the transition to automation might be at fundamental odds with the indigenous authenticity of the method. The method proposed here, after all, originates from an ancient art of seafaring that utilized abstraction in combination with raw physical sensations alone, which is why it is logical that the same level of primitivism be tied to its implementation as well.

Regarding the cell selection criteria, highly symmetrical cells were not analyzed for their positioning with respect to jur in okme squares; only cells that appeared pronouncedly asymmetrical were included in the analysis. The cells forming overly confluent conglomerates were also excluded because their shapes could hardly be discerned under those conditions. The location and the orientation of the points K and L with respect to the cell boundaries would provide for a different metric, which was not tested in this study. Another metric deemed meaningful to probe was that of fitting a greater portion of the cell, including the whole nucleus, within the central area, M, after which the location of the jur in okme squares at a certain distance from the cell was measured and compared geometrically against different sample groups, in which situation the similarity between the cells and the atolls appeared even more realistic. One such comparison did not yield a statistically significant difference between the control and the treated populations in terms of the proportion of the jur in okme squares overlapping with cells in the image, but it is possible that different considerations of the neighboring cells within which the jur in okme squares fall in this metric might bring about more significant differences between these two populations.

**Figure 6:**
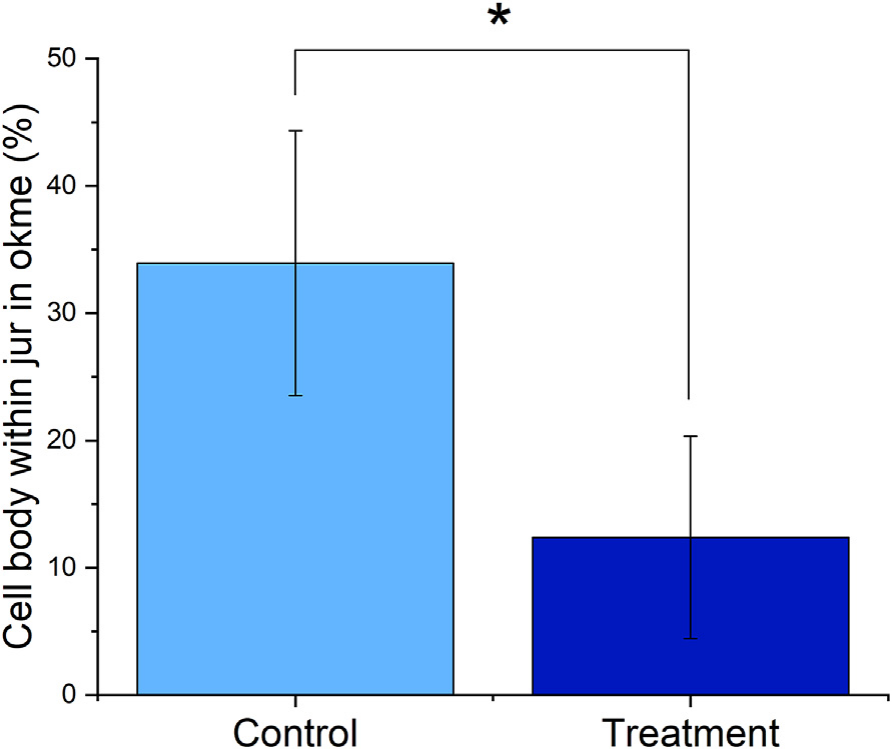
Comparison between the average proportion of jur in okme squares lying within the boundaries of the control cells and cells subjected to the treatment. Data points represent averages, while error bars represent standard deviation. Statistically significant difference (p < 0.05) between the data point corresponding to the control group and the data point corresponding to the treatment group is denoted with an asterisk.

A systematic comparison of the positioning of jur in okme portions of the mattangs superimposed over the precisely oriented cells depending on whether they were treated or not demonstrated a distinct trend supported by a statistically significant difference (Figure 6). Namely, the proportion of the cell body confined to the jur in okme squares of the mattangs was markedly lower in cells subjected to the treatment compared to the control cells. While 12.4 ± 7.9% of the jur in okme squares in mattangs superimposed over the treated cells was covered by the cell surface, this amount was almost three times higher in mattangs superimposed over the control cells. One reason for the observed effect between the jur in okme square positioning in control cells and the treated cells was initially thought to be due to the difference in the area covered by the cell nucleus compared to the total cell area between these two cell populations. This was considered especially likely since cancer cells, like those utilized in this study, are often diagnosed based on the graded increase in nuclear sizes compared to their healthy counterparts [44]. However, both the control and the treated cells were an identical osteosarcoma clone and no difference in the size of the nucleus relative to the size of the cell was consistently noticed. Therefore, it is more likely that the subtle change occurring due to the treatment at the level of spatial ordering of the cells in culture may affect their morphologies through modified cell signaling and gene expression, or through altered microcurrents in the cell culture medium in analogy with the swells interacting at and around the atolls of the Marshall Islands. If so, then the lower percentage of the cell bodies found within the area of intense current intersections as per the mattang charts may suggest that the treatment makes the cells slightly more susceptible to these microcurrents and prone to draw their boundaries inward under their influence. This explanation, of course, implies the acceptance of the direct relevance of mattangs in estimating the hydrodynamic states of the medium surrounding the cell, which is a bold hypothesis to think of, let alone employ as a supposedly true premise. However, from the fundamental thermodynamic standpoint, the periodic variations in osmotic pressure arising from the metabolism of nearly a million cells per cm^2^ of the cell culture dish must produce very fine streams in the liquid medium, which might constructively or destructively interfere with one another and exert an effect on the cell morphologies. The techniques for detecting the presence of these microcurrents and verifying their effect on the cells, however, are purely hypothetic at this point. The enigmatic mechanism underlying these effects notwithstanding, the Marshallese mattang is proven here as a potentially useful tool for estimating the cell fate in response to a treatment at its early stages, when the effects of it may still be reversible.

### 3.3 Correlation with the Carolinian etaks

**Figure 7:**
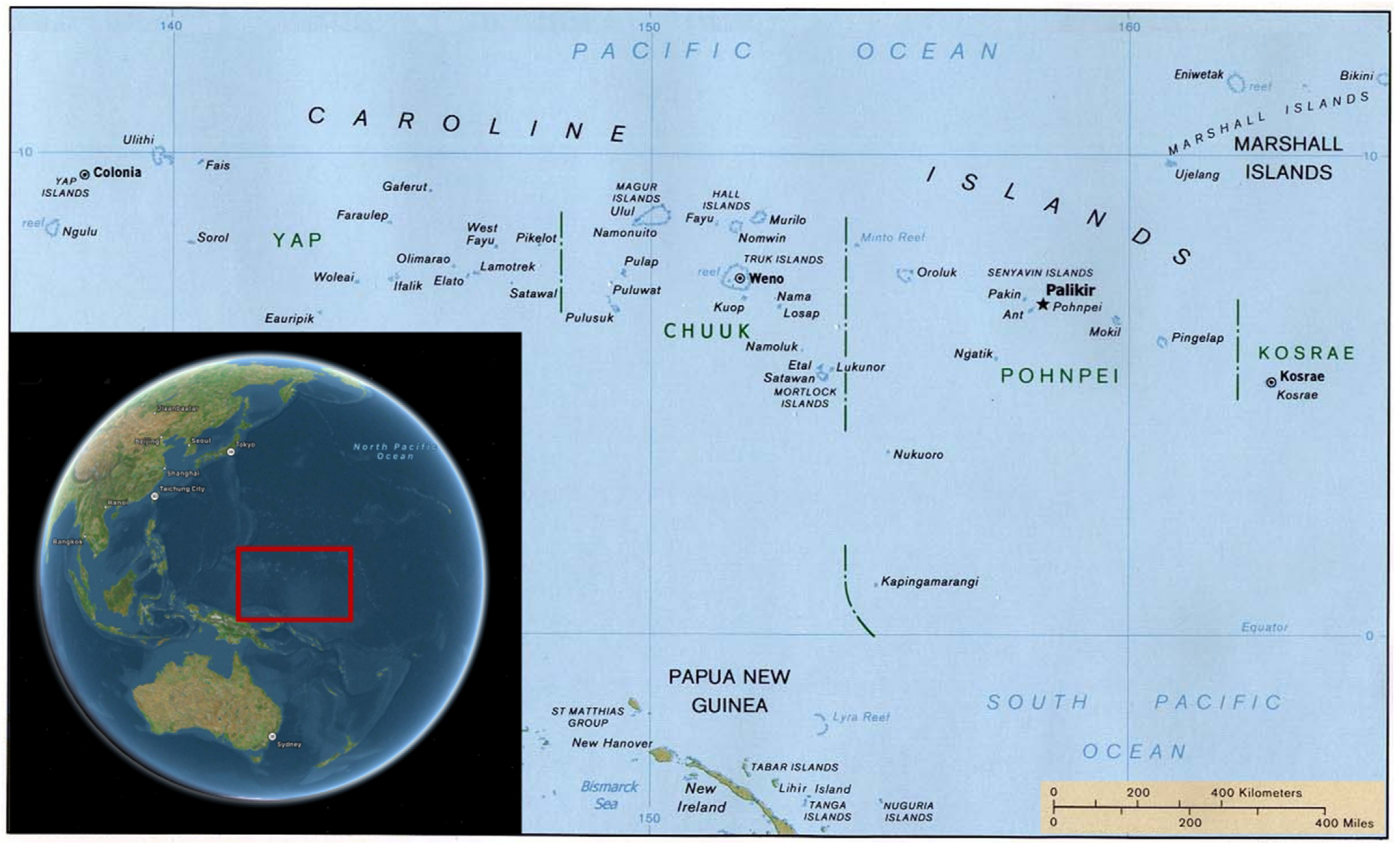
Map of the Caroline Islands in the Pacific and their location on the globe with respect to the Eurasian and Australian continents (inlet).

All Pacific islanders were impelled throughout the centuries of sea voyaging to devise navigational strategies more sophisticated than mere dead reckoning. Used by sailors in the absence of any other methods, this most basic of all navigational techniques involves the estimation of the position of the vessel based on the course and the speed corrected approximately for the effects of the leeway and the current. Among all the traditional schools of navigation in Oceania, the Carolinian art of navigation is considered to have provided the greatest level of sophistication compared to dead reckoning [45]. Like the Marshall Islands, the islands and atolls in the Carolines are very dispersed (Figure 7), with the distances between the coasts of the nearest neighboring coral atolls or islands averaging at over 200 miles. Therefore, the indigenes’ voyaging between these shores was in dire need of a solid and reliable navigational system, which the Carolinians did devise and continue to refine over the two millennia of the existence of human settlements on these islands.

The key aspect of the Carolinian navigational system consists in demarcating etak reference islands, which lie sideways from the main trajectory between the point of origin and the destination of the voyage [46]. This etak reference island is characteristic because it eclipses or lies directly under one star at the point of origin, another star at the destination and one or more stars during the journey, where the number of stars eclipsed or positioned right over this reference island, m, is related to the number of etaks, n, with the following simple relation:

n = m + 1

**Figure 8:**
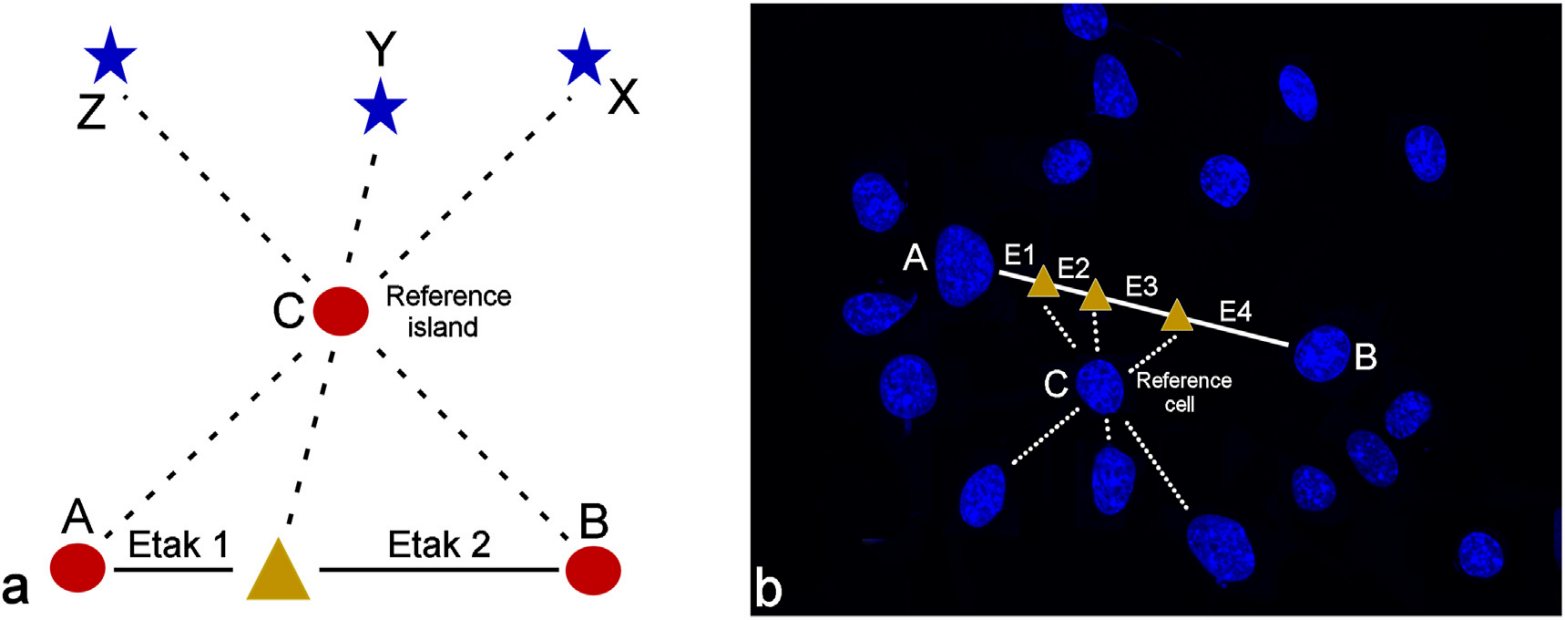
Illustration of the Carolinian navigational technique based on the concept of the etak and involving a voyage from the island A to the island B using the island C as the etak reference island (a). At the point of origin, the star X is either eclipsed or positioned right over the reference island C. The end of the first segment (i.e., etak) of the voyage is marked when the star Y is either eclipsed or positioned right over the reference island C. As the island B, the destination of the voyage, is reached, the star Z gets eclipsed or positioned right over the reference island C, marking the end of the final segment (i.e., etak) of the journey. This method is converted to the cellular context by substituting stars with neighboring cell nuclei. The sideways-positioned cell nucleus lying nearest to the straight line connecting the two cells selected as the point of origin (A) and the destination (B) is denoted as the reference cell (C) (b). Three cell nuclei in the exemplary image (b) get eclipsed by the reference cell nucleus during the voyage from the center of the cell A to the center of the cell B, meaning that the number of etaks in this case is 4. The average number of etaks per sample is measured on each pair of cells in an image connectable by a straight line, where at least one cell nucleus in the image is eclipsed by the reference cell lying closest to the middle of the line connecting the cells.

Therefore, a journey with two etaks will have one star eclipsed or positioned directly over the reference island midway through the journey, as it is illustrated in Figure 8a; the journey with three etaks will have two stars eclipsed by or positioned directly over the reference island during the journey; and so on. Albeit seemingly simple, this navigational method is interpreted with a great dose of ambiguity, similar to that tied to the interpretations of the mattang. These uncertainties start with the very nature of the etak reference point [47]. For one, it is still debated whether these reference islands were real or imaginary. In some scripts, they are said to represent islands posed laterally, but halfway between the point of origin and the destination, offering potential emergency landing sites in case the journey goes awry due to storm, sickness or damage to the boat. Other scripts, however, assure that the nonexistent islands, such as the mythical “vanishing islands” of Kafeŕoor and Fanuankuwel, were regularly chosen as etak reference points by the voyagers, thus questioning the correctness of the hypothesis of emergency islands as reference points [48]. Most voyages, in fact, were such that no single reference island could be held on the horizon for the duration of the sail. Therefore, it is often asserted that the nature of this island must have been imaginary [49], moving together with the vessel and helping the navigator steady the perception of the course determined mostly by the sensing of the currents and the winds, and aligning with the stars. Untrained to give an aerial perspective to his mental constructs of the ocean, the Carolinian navigator may have relied on the visualization of the etak reference island off to the side of the direction of the sail as a sort of anchor for achieving this broader spatial perspective.

**Figure 9:**
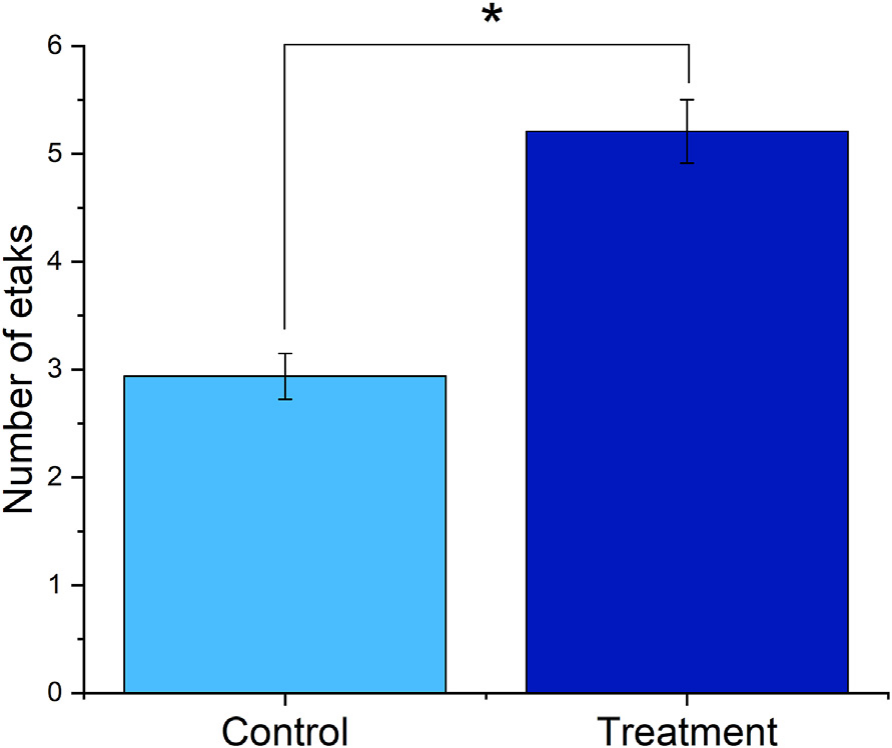
Comparison between the average number of etaks per image for the control cells and for the cells subjected to the overnight treatment with superparamagnetic composite nanoparticles. Data points represent averages, while error bars represent standard deviation. Statistically significant difference (p < 0.05) between the data points corresponding to the control group and the data points corresponding to the treatment group is denoted with an asterisk.

A correlation was attempted to be established between this ancient Carolinian navigational art based on the concept of the etak and the spatial ordering of mammalian cells in response to the aforementioned treatment with the superparamagnetic nanoparticles. To do so, individual cells in the culture, adhering to the bottom of the cell culture dish, were taken as analogous to the islands. The use of stars for the alignment purposes in cell culture is challenging because of the ambiguity of their orientation with respect to the cells and the presumed irrelevance of cardinal directions to the cell culture. For this reason, other cells in the distance, whose view gets covered by the etak cell chosen as the reference point, were used in lieu of stars. Because the type of cell culture utilized in this study was planar, only the eclipse of cells by the etak reference cell counted toward the number of etaks. Regarding the choice of this reference cell, it was not ambiguous; rather, the cell nucleus lying nearest to the straight line connecting the nucleus selected as the point of origin and the nucleus selected as the destination was used as the etak reference cell. Hence, as shown in Figure 8b, a hypothetic journey from the cell A to the cell B using the cell C as the etak reference cell would lead to the eclipse of three different cells by the reference cell during the voyage, meaning that the number of etaks, n, is 4. To make this method feasible, the cells were stained for their nuclei only and the inter-nuclear distances were approximated as the intercellular ones. In other words, both the line connecting two cells voyaged between (solid line in Figure 8b) and the line of eclipse (dashed line in Figure 8b) could pass through the cytoplasm of other cells, but not through their nuclei. With this methodology, it was counted how many etaks on average it would take to traverse the path between each pair of cells in an image connectable by a straight line, for which at least one cell nucleus in the image would be eclipsed by the reference cell. These averages were further averaged for no less than 3 images in 3 different experimental replicas and compared statistically between the control sample group and the treated sample group. The etak counts were made on fluorescent images displaying distributions of cell nuclei at an identical magnification and containing 30–40 cells per image. As per the results of this comparison shown in Figure 9, at 5.21 ± 0.29%, the average number of etaks was significantly higher in the treated cell population than in the control one, where the average number of etaks was 2.94 ± 0.21%.

**Figure 10:**
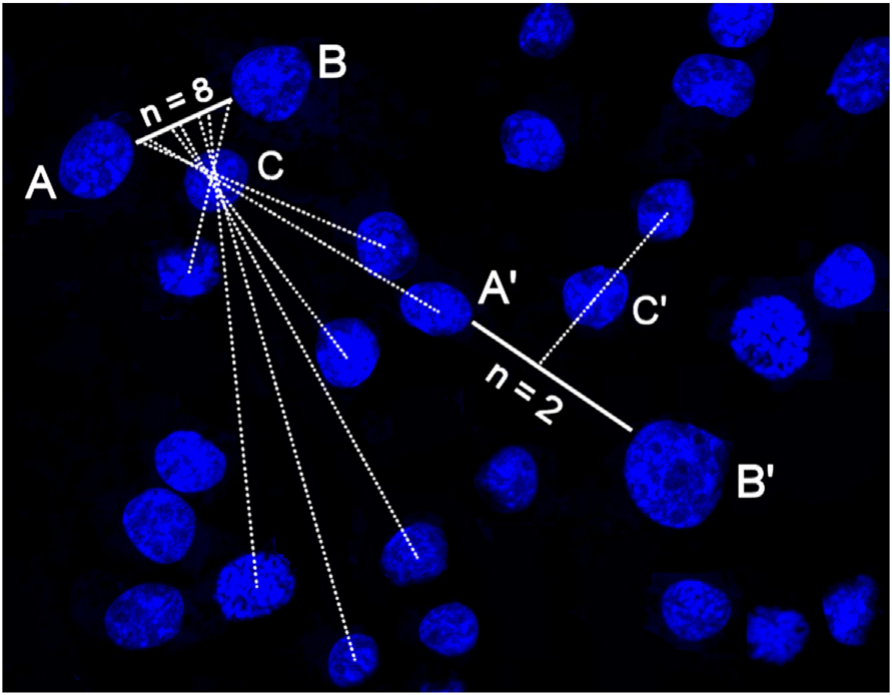
Distance between cells, the nuclei of which are stained in blue, is not directly proportional to the number of etaks, n, as illustrated by a relatively short distance between cells A and B divisible to 8 etaks (n = 8) compared to a longer distance between cells A′ and B′ divisible to only 2 etaks (n = 2).

This significantly higher etak number for cells in the treated population as compared to the control may be a consequence of the earlier detected dispersal of the cells after the treatment and their yielding more regular distance matrix patterns than the control, untreated populations [50]. The latter effect may be explained by the natural tendency of cells that have embarked on the apoptotic path as the result of the therapy or other stimuli to individuate and estrange themselves from the cellular community, an effect that at the spatial level manifests itself as equidistant or semi-equidistant separation. Indeed, before their membrane leaks and disintegrates and they lose integrity and clump into an indistinct mass, freshly dead cells in culture dishes do tend to be found at relatively regular distances from one another [51]. In addition, one may be tempted to assume here that in a sample containing a stochastic distribution of cells, the larger distances between them would translate to larger etak numbers, but this has not been so. As exemplified in Figure 10, very short distances could often yield extremely high etak numbers, whereas very large distances could lead to minimal etak numbers. Therefore, the reasons for this significant of a difference between the two populations must be sought in the subtle specificities of the cell spatial ordering. Detecting this difference with a method mimicking the Carolinian seafaring navigation technique indirectly demonstrates that the cell-cell interaction precedes any adverse effects visually detectable in individual cells, which is an interesting insight in itself, independently of the ethnoscientific subject of this study. A tool enabling the detection of such early signs of adversity based on visual cues that fall in the domain of translational symmetry [52] would present an essential addendum to the range of bioimaging tools used for *in situ* monitoring of the response of cells and tissues to therapies in the state-of-the-art medical labs of the day. Finding the fundamental principles for building one such tool in the scientific tradition harbored by the Micronesian mariners of the past expands our horizons beyond even the most mythical and imaginary islands used by the Carolinians in their voyages. The repercussions of one such successful search fall back with benefits to the very same political grounds that motivated the search in the first place, hoping to heal everything that is inhumane and corrupt on these grounds and turn them into sources of newly awakened humanness where differences and diversities would be appreciated and allowed to grow instead of being suppressed and erased.

## 4 Conclusion

Two distinct traditional Micronesian methods for navigation across the ocean were assessed as bridges for correlating (a) the interaction of oceanic swells near atolls and islands with the way microcurrents may shape the morphology of cultured cells, and (b) the spatial arrangement of cultured cells with the canoe voyaging from one island in the ocean to the next. Both of these methods were shown as effective for enabling the prediction of the cell fate at early time points in the treatment, when the adverse effects were yet to appear at the levels of cell morphology, proliferation rate and confluence. Specifically, the comparison with the Marshallese navigational technique demonstrated that the mattang chart, the most fundamental and theoretical of devices used in the Marshallese maritime school, could be used to measure subtle morphological changes occurring in cells due to the treatment delivered in the form of superparamagnetic nanoparticles and designed to provide a gradual decrease in the cell viability over time. The cells subjected to the treatment appeared to have withdrawn their bodies from the key areas of the superimposed mattang, namely the square-shaped jur in okme zones of intense swell interaction activity. Given that the cytoskeletal microfilaments defining the features of control cells were largely filling up this area, this metric proved useful for deducing the course of the treatment at its early stages, when more severe morphological deformities, including cell rounding and detachment, were yet to take place. The same deduction was proven feasible with the use of a Carolinian navigation tool based on the concept of the etak. In that case, the distances traversable between cells in a population subjected to the treatment were divisible to a significantly higher number of etaks than the same distances in the population of control cells. Therefore, treating cells and their nuclei as analogous to Pacific atolls navigable to and fro with the use of imaginary microscopic canoes and the navigational principles native to the Marshall and the Caroline Islands proved as a wildly imaginative, but also quite effective cell fate prediction approach, which various branches of biomedical science could make use of. If so, this would show that the end is not the end, and even when the last traces of a culture are erased, it may continue to live and inspire.

These practical benefits notwithstanding, this has been, first and foremost, a conceptual study performed with a goal to spark the interest in studying stringently the use of the Marshallese, Carolinian and other ancient navigational tools and ethnoscientific inventions as potential addenda to the broad repertoire of techniques used in biomedical and other sciences to combat some of their greatest challenges. The satisfaction for engaging in these successful ethnoscientific voyages of the intellect are immense, as they bring about a sense of unity and connectedness of things near and far, past and future, obscure and trendy. At the same time, a voyager, amidst this bliss and a suddenly awakened sense of belonging everywhere, feels alone. Alone he is in a world that zooms by with the zeal to jump on the bandwagon and bandwagon only, neglecting to look at the ethnic scientific traditions long gone and explore them for intellectual treasures that they may hold within. Like the Lolleplaplapian youth drifting in the bottom of his canoe, feeling the rocking of waves and wavelets, thinking about an island that will soon no longer be, about the past and the future that will swallow the past in no time and sink it into a darkest limbo, our voyager drifts in this open sea carrying him toward the islets of new methods and perspectives that lie out of sight, wholly unforeseen. With the loneliness of a solo canoeist in the open ocean seated deep inside his mind, with no land in sight, he is destined to wander, endlessly.

The ocean is so big. And the Lolleplaplapian is so small. A wave will soon come and he will be under, but the vanishing will not be left unnoticed. The beauty of science is to make the small big and even smaller even grander. These words, if heard, may bring about the message of these forgotten lands to this brave new world busying itself with the progress along a narrow paradigmatic plane, paying no heed to worldviews from the past and present that differ fundamentally from it. Only those who have, themselves, felt swamped by such and similar waves would appreciate the value of disappearing, downward spiraling cultures and ways of life and find meaning in aspirations to resurrect and engage them into a cross-disciplinary union with what is considered hip and trendy. To feel swallowed by the waves, to feel going under for good, to feel destined for the darkest depths of oblivion, to be poorer than the poor, as it were, thus, can be a blessing, for it opens our eyes to a plethora of worldviews different than ours, but equally potent as them.

Deprivation and loss, in the end, can be the drivers of a more creative and trailblazing research than that rooted in resourcefulness and wealth, which usually proceeds in inert programmatic compliance with the fad. This, in the end, has been one in a series of studies that sprang from economic poverty [53, 54, 55, 56], from meager resources for research and from the wish to liberate an inventive mind from the shackles of dependence on material abundance and prove that it, at its best, rules over matter.

And now, as the end has rolled around and the voyager is deep under the waves, streaming toward the bottom of the ocean, what is left to be said? His mission seems to have failed, but his heritage might continue to live. If it only, like the ancestral Micronesian science elaborated here, gets rediscovered by a gentle heart and be put under a microscope.

## Declarations

### Author contribution statement

Vuk Uskoković conceived the study and designed the experiments; analyzed and interpreted the data; wrote the paper.

### Funding statement

This research did not receive any specific grant from funding agencies in the public, commercial, or not-for-profit sectors.

### Data availability statement

Data will be made available on request.

### Declaration of interest's statement

The authors declare no conflict of interest.

### Additional information

No additional information is available for this paper.
